# Retinal ganglion cell interactions shape the developing mammalian visual system

**DOI:** 10.1242/dev.196535

**Published:** 2020-12-07

**Authors:** Shane D'Souza, Richard A. Lang

**Affiliations:** 1 The Visual Systems Group, Cincinnati Children's Hospital, Cincinnati, OH 45229, USA; 2 Center for Chronobiology, Abrahamson Pediatric Eye Institute, Division of Pediatric Ophthalmology, Cincinnati Children's Hospital, Cincinnati, OH 45229, USA; 3 Molecular and Developmental Biology Graduate Program, University of Cincinnati, College of Medicine, Cincinnati, OH 45229, USA; 4 Division of Developmental Biology, Cincinnati Children's Hospital, Cincinnati, OH 45229, USA; 5 Department of Ophthalmology, University of Cincinnati, College of Medicine, Cincinnati, OH 45229, USA

**Keywords:** Retinal ganglion cells, Visual system, Non-autonomous, IpRGCs, Cell-cell interactions, Retina

## Abstract

Retinal ganglion cells (RGCs) serve as a crucial communication channel from the retina to the brain. In the adult, these cells receive input from defined sets of presynaptic partners and communicate with postsynaptic brain regions to convey features of the visual scene. However, in the developing visual system, RGC interactions extend beyond their synaptic partners such that they guide development before the onset of vision. In this Review, we summarize our current understanding of how interactions between RGCs and their environment influence cellular targeting, migration and circuit maturation during visual system development. We describe the roles of RGC subclasses in shaping unique developmental responses within the retina and at central targets. Finally, we highlight the utility of RNA sequencing and genetic tools in uncovering RGC type-specific roles during the development of the visual system.

## Abstract (precis)

**Summary:** This Review summarizes the extensive roles of retinal ganglion cell interactions in shaping cell migration, vascular development, circuit formation and behavior, before the onset of vision in mammals.

## Introduction

**Fig. 1.**
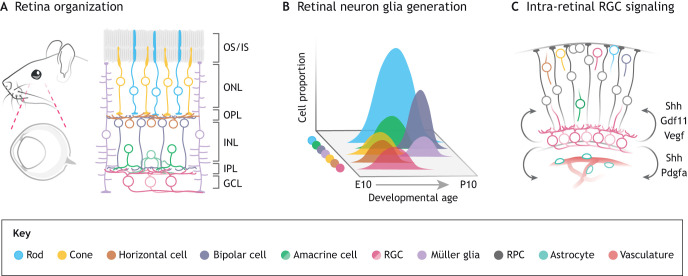
**Retinal architecture, cell generation and intra-retinal signaling during retinal development.** (A) The mammalian neural retina highlighting photoreceptors (rods, cones), interneurons (horizontal cells, bipolar cells and amacrine cells), projection neurons (RGCs), Müller glia and retinal progenitor cells (RPC) structured into three nuclear/cellular layers (ONL, outer nuclear layer; INL, inner nuclear layer; GCL, ganglion cell layer) and two neuropils (OPL, outer plexiform layer; IPL, inner plexiform layer). (B) Developmental generation of mouse retinal cell types (based on Young, 1985) depicting both the sequence of generation and relative proportion of each cell type. (C) Interactions between immature RGCs and other developing cell types in the context of retinal development. Arrows depict RGC-derived factors described in the text. OS/IS, outer segment/inner segment of photoreceptor cells.

The retina serves as the visual gateway to the world around us. This light-sensitive neural tissue is located in the posterior of the eye (Fig. 1A) and has evolved to detect photons, extract visual features and convey this information to the rest of the brain (Baden et al., 2020). Together, the eye and all brain regions involved in processing retinal input form the visual system (Seabrook et al., 2017). Through the complex and integrated function of multiple cell classes in the retina (Fig. 1A), we are capable of sensing, experiencing and responding to visual scenes that range from subtle and static to vivid and dynamic.

Photoreceptors (rods and cones) express photopigment proteins and detect light. These photoreceptors signal to bipolar interneurons, which then provide excitatory input to retinal ganglion cells (RGCs) (Fig. 1A). Horizontal cells provide inhibitory input to photoreceptors, whereas amacrine cells inhibit RGCs and bipolar cells (Masland, 2012). RGCs have axons that extend through the optic nerve and convey information from the retina to numerous targets in the brain – shaping not only visual perception, but also our physiology and circadian behavior (Schmidt et al., 2011; Baden et al., 2020). Unraveling how visual processes take place requires an understanding of the cell diversity, mapping their connections, deciphering communication patterns and determining how each cell is generated. In addition, it is vital to determine how transient developmental interactions between cells shape the visual system. By systematically addressing these various aspects of the visual system, we can slowly begin to tease apart the complexities of visual mechanisms.

RGCs, the sole projection neurons of the retina, have been well-studied for their diversity, connections, signaling and development. Between electrophysiological profiling and single-cell transcriptomics, this class can be further divided into 30-46 distinct types in mice (Baden et al., 2016; Tran et al., 2019). Each RGC type optimally encodes unique features of the visual scene (contrast, motion, color, etc.), and projects to diverse ‘central targets’ in the brain that decode each feature (Dhande et al., 2015). Some RGC types express the photopigment melanopsin (Opn4), conferring them with direct photosensitivity (Berson et al., 2002; Hattar et al., 2002). Collectively, these multiple photosensitive RGCs are referred to as intrinsically photosensitive RGCs (ipRGCs) and make up a subclass representing six unique RGC types (M1-M6; reviewed by Sondereker et al., 2020).

Interestingly, RGCs also represent one of the first cell classes generated during retinal development (Fig. 1B) (Young, 1985) and their cellular diversity is established by postnatal day (P) 5 (Rheaume et al., 2018; Tran et al., 2019), before eye opening in mice. Their numerous interaction partners in the brain and retina, in addition to their early generation, indicate that RGCs could regulate developmental events along the visual pathway. Over the last two decades, evidence has emerged indicating that RGCs function as more than visual feature detectors and information conduits. Although sparse (∼2.7% of all retinal cells) (Young, 1985), RGCs serve as a developmental nexus for the visual system. By interacting with migrating retinal neurons, retinal and vascular progenitors, astrocytes and central targets, they influence the course of visual system development, well before the visual experience.

In this Review, we provide an overview of the evidence suggesting that RGCs play a developmental role in mammalian visual system development. When applicable, we describe key concepts gleaned from non-mammalian vertebrate models. We describe how early RGC activity, structure and secreted factors establish structural, molecular and physiological properties within the visual system. When available, we highlight data that implicate particular RGC subclasses (e.g. ipRGCs) as modulators of retinal and central development, and how this influences behavior. Finally, we describe how next-generation tools (bioinformatics and genetics) can be applied towards understanding how RGCs contribute to development of the retina, brain and mammalian behavior.

## The roles of RGCs in retinal development

The developmental influence of RGCs can be categorized into two types: activity-dependent input (in which RGCs depolarize and release neurotransmitters) and activity-independent input (secreted factors or structural aspects of the cell). In this section, we describe how these forms of early ‘RGC input’ influence the developing retina, optic tract and optic chiasm (Fig. 1C).

### RGC-derived factors modulate progenitor proliferation and differentiation

The neural retina (Fig. 1A) is derived from a pool of multipotent retinal progenitor cells (RPCs). These cells give rise to all retinal neurons and Müller glia, largely relying on intrinsic regulation (such as transcriptional regulators and chromatin state) and extrinsic cues (paracrine factors) to guide specification into one of seven post-mitotic cell classes (Cepko, 2014). Two models of intrinsic regulation have been proposed to describe how progenitors generate each of these classes in a temporally defined sequence (Fig. 1B). The deterministic model suggests that intrinsically different RPCs are competent to generate specific groups of classes (Turner et al., 1990; Trimarchi et al., 2008). By contrast, the stochastic model suggests that RPCs are equipotent and, through a probabilistic process, generate cell classes in the quantities and order observed during retinal development (Cayouette et al., 2003; Gomes et al., 2011; He et al., 2012). Our current understanding, however, suggests that both modalities of development likely occur in the retina (Cepko, 2014). In addition to differentiation of these classes, RPCs must also self-renew to generate cells in appropriate quantities. Although intrinsic factors appear to be a major driver of clone size (and thus number of RPC-derived retinal cells) (He et al., 2012), there are documented circumstances in which RGC-derived signals (extrinsic cues) shape the generation of specific cell classes and cell quantity during development.

Early experimental approaches using dissociated or explanted rodent retina cultures, heterochronic mixing (combining cells of different developmental ages) and media supplementation suggest that post-mitotic cells have the capacity to influence RPC differentiation (Watanabe and Raff, 1990, 1992; Altshuler and Cepko, 1992; Waid and McLoon, 1998). The presence of RGCs (in culture) prevents progenitors from adopting the RGC fate (Waid and McLoon, 1998), and the responsible RGC factor(s) appears to be diffusible as opposed to juxtacrine. The morphogen sonic hedgehog (Shh) was found to be expressed by developing RGCs, while its cognate receptor (patched) is expressed by RPCs (Jensen and Wallace, 1997; Zhang and Yang, 2001). These discoveries position this axis as a feedback pathway that titrates RGC production in the retina. More recent studies have firmly established a developmental role for RGC-derived Shh. Murine Shh is initially expressed by RGCs of the central retina at embryonic day (E) 12 (Wang et al., 2005). In fish and mice, Shh expression expands in a central-to-peripheral wave that closely mirrors, but lags behind, the wave of RGC generation (Neumann and Nuesslein-Volhard, 2000; Wang et al., 2005). RGC-derived Shh serves two major roles during retinal development: promoting proliferation of RPCs, and simultaneously suppressing RGC generation. Loss of *Shh* in the peripheral retina leads to reduced RPC proliferation, increased RGC production, and failure of later-born cells to be generated (Wang et al., 2005).

Another diffusible factor, vascular endothelial growth factor (Vegf), is expressed by RGCs, and its receptor (Flk1; also known as Kdr) is expressed by endothelial cells (Yamaguchi et al., 1993) and RPCs (Yang and Cepko, 1996). This expression pattern is conserved in chicken, in which secreted Vegf suppresses RGC-genesis and promotes RPC proliferation, reminiscent of Shh function in the developing retina (Hashimoto et al., 2006). RGC-specific ablation experiments (Mu et al., 2005), and mice with RGC loss (Gan et al., 1996; Brown et al., 2001), also complement the claim that RGCs are a source of proliferative cues. By contrast, not all RGC-released factors influence RPC proliferation. RGC-derived growth differentiation factor 11 (Gdf11) inhibits progenitors from adopting the RGC fate without altering cell cycle dynamics. Instead, it is thought that Gdf11 temporally restricts the expression of key RGC-competence factors (Kim et al., 2005). The gene encoding one such transcription factor, *Atoh7*, has been extensively studied for its requirement in RGC-genesis through the induction of an RGC-fate transcriptional program (Liu et al., 2001; Wu et al., 2015). In several vertebrate classes assessed (Brown et al., 2001; Kay et al., 2001; Liu et al., 2001), including humans (Prasov et al., 2012), mutations in *Atoh7* or its regulatory elements lead to an almost complete loss of RGCs, with additional pathologies associated with the eye (Ghiasvand et al., 2011; Prasov et al., 2012; Kondo et al., 2016; Atac et al., 2019). In addition, recent data suggest that Atoh7^+^ lineage RGCs non-autonomously promote the survival or generation of Atoh7^−^ lineage RGC types (Brodie-Kommit et al., 2020 preprint). Thus, RGCs appear to regulate their own production by feeding into RPC transcriptional programs via paracrine signaling.

Although it is clear that RGCs enhance progenitor proliferation through secreted factors, it is debated whether the RGC-to-RPC axis influences fate determination. Studies involving gene deletions of key factors in RGC development and production [*Shh*, *Atoh7*, *Brn3b* (*Pou4f2*), etc.] argue that the RGC-to-RPC signaling axis, in addition to regulating proliferation, differentially influences the production and survival of certain cell classes (Wang et al., 2002, 2005; Moshiri et al., 2008; Bai et al., 2014). Studies using intersectional genetics to specifically ablate RGCs (*Six3-cre; Brn3b^z-DTA^*) suggest that all non-RGC cell classes are produced in correct proportions but, overall, retinal cell number is greatly reduced (Mu et al., 2005). However, both forms of manipulation have limitations that make it difficult to interpret their meaning for fate-determination. For example, *Shh* expression is not restricted to RGCs during development – murine amacrine cells and cones also express Shh at later developmental stages (Jadhav et al., 2006). Thus, alterations in germline *Shh* knockouts may reflect alternative sources of Shh. In addition, the kinetics of RGC death in the *Six3-cre; Brn3b^z-DTA^* line is not fully understood, making it possible that transient RGCs are sufficient to shape fate determination. Further work is required to establish RGCs as bona fide modulators of fate determination in the retina.

### Non-autonomous regulation of lamination via RGCs

During development, retinal neurons must be generated in the appropriate numbers, but must also locate their synaptic partners in order to effectively encode the visual scene. In the first few postnatal weeks, the processes of synaptic formation, pruning and circuit establishment occur through cellular activity (Demarque et al., 2002; Goldberg et al., 2002), expression of attractive or repulsive cell-surface proteins (Sanes and Zipursky, 2010; Missaire and Hindges, 2015; Sanes and Zipursky, 2020), and release of neurotransmitters (Morgan et al., 2008; Kerschensteiner et al., 2009; Blankenship and Feller, 2010). In particular, these events take place in the inner plexiform layer (IPL) (Fig. 1A), a synaptic ‘highway’ in which bipolar axons meet RGC and amacrine dendrites in distinct laminar sublayers. Here, the visual field is preprocessed by the retina and separated into parallel channels based on information content, before being conveyed to the brain (Sanes and Zipursky, 2010, 2020).

**Fig. 2.**
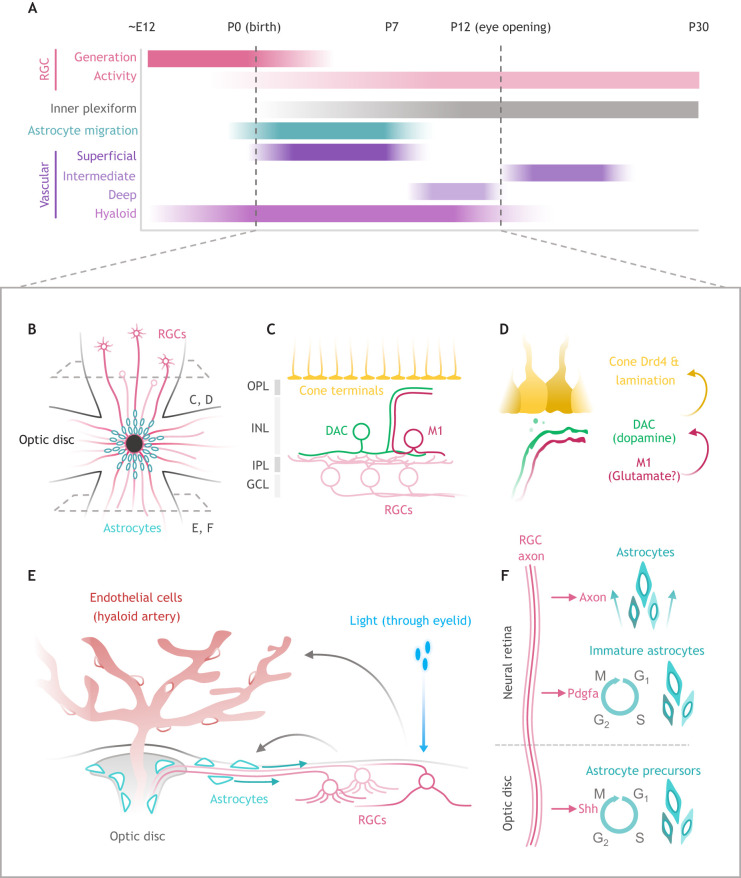
**RGCs interact with non-synaptic partners to guide lamination and vascular development.** (A) Timeline of retinal ganglion cell (RGC) generation and activity relative to development of the inner plexiform, migration of astrocytes and vascular growth/regression in the retina. (B) Whole-mount schematic representation of a developing retina depicting astrocyte migration from the optic disc. (C) Dendrites extending from M1 intrinsically photosensitive retinal ganglion cells (M1) and dopaminergic amacrine cells (DACs) extend beyond the appropriate synaptic location in the inner plexiform layer (IPL) towards the outer plexiform layer (OPL), where cone photoreceptor synaptic terminals are located. (D) Communication between the intrinsically photosensitive retinal ganglion cell (ipRGC) and DAC leads to dopamine release, activation of D4-type dopamine receptor (Drd4) on cones to restrict lamination of the cells to the outer nuclear layer (ONL). (E) Immature RGCs interact with migrating astrocytes and the hyaloid artery through RGC structure (axons) and RGC activity (photoreception), respectively. (F) Timeline of the influence of RGC-derived factors and structure on the glial/astrocyte lineage in the optic disc and to neural retina. GCL, ganglion cell layer; INL, inner nuclear layer.

IPL laminar structure is generated via attractive interactions between presynaptic and postsynaptic cells (Yamagata et al., 2002; Yamagata and Sanes, 2008). Given the early generation, migration and activity of RGCs (Young, 1985) (Fig. 2A), one might expect RGCs to guide axonal and dendritic lamination of the later-born bipolar and amacrine classes (Fig. 1B). There is some evidence for this in the zebrafish retina, in which RGCs are transiently required for appropriate amacrine dendrite lamination in the IPL (Kay et al., 2004). In mice, as we have discussed, studies using genetic ablation or loss of RGCs reveal that ganglion cells may not be required for general targeting or lamination of neuronal processes in the mammalian IPL (Mu et al., 2005; Moshiri et al., 2008). It is possible that, within a circuit, interacting inner retinal neurons mutually restrict lamination patterns. For example, the ramification of ON-OFF direction-selective RGC dendrites is dependent on the homophilic interaction between cadherins expressed by these cells and their synaptic partners, the starburst amacrine cells (SACs) (Duan et al., 2018). Although it is clear that lamination requires the interaction between multiple cell types, RGCs do not appear to be a major influence during construction of the IPL. Further research into specific circuits and RGC types will help determine the role of RGCs in synaptic lamination.

Interestingly, one type of RGC, the M1 ipRGC, extends its dendrites beyond the IPL during development, and is poised to regulate more than just its synaptic partners. ipRGC types are defined by their dendritic structure and lamination pattern, soma size, melanopsin expression and projections to central retinal targets (Sondereker et al., 2020). M1 ipRGCs terminate their dendrites in the outer IPL (Provencio et al., 2002), but during postnatal development, they extend their dendrites towards the outer retina (Fig. 2B,C) making them ‘biplexiform cells’ (Renna et al., 2015). These atypical projections, termed outer retinal dendrites (ORDs), are transient and their distribution is limited to the dorsal retina (Sondereker et al., 2017). The close proximity of these dendrites to cone terminals suggests a potential interaction between these two classes of photoreceptor. Indeed, early activity of murine ipRGCs is necessary to restrict cone photoreceptors to the outer retina (Tufford et al., 2018). This occurs through an indirect mechanism: ipRGC ORDs contact dendrites of dopaminergic amacrine cells (DACs) and through potential regulation of dopamine release, cones are restricted to their appropriate layers (Fig. 2D). In addition, cone cell bodies are further restricted to the apical aspect of the outer nuclear layer (ONL) via light- and activity-independent input of ipRGCs (Fig. 1A; Tufford et al., 2018). Taken together, these studies suggest that, as a class, RGCs do not shape their local synaptic plexus (i.e. the IPL), but signaling of an RGC subclass (ipRGCs) restricts non-synaptic partners to their appropriate location.

### Developing astrocyte and vascular networks require immature RGC input

RGC-mediated regulation of visual development is not limited to neuronal cells of the retina (Fig. 2A). As RGCs are generated, their axons project towards the optic disc (Fig. 2B,E), fasciculate to form the optic nerve, and travel great distances to contact central targets. At the optic disc, RGCs are poised to interact with cells of the optic stalk, astrocytes and the hyaloid vasculature – a transient structure that serves as the primary nutrient source for the developing eye (Fig. 2E). Cues originating from developing RGC axons appear to stimulate proliferation of astrocyte precursors (Burne and Raff, 1997). Surprisingly, in addition to its local role in the neural retina, RGC-derived Shh is transported down the axon and promotes astrocyte precursor and optic stalk progenitor proliferation in the optic disc (Fig. 2E,F; Wallace and Raff, 1999; Dakubo et al., 2003). Overall, RGC-derived Shh serves similar roles across the developing visual system, promoting proliferation of retinal, astrocyte and optic stalk progenitor cells.

Astrocytes enter the retina through the optic disc, migrate radially and provide a framework for endothelial cell growth and vascular development (Fruttiger, 2007; Sun and Smith, 2018). Radial migration of astrocytes depends on paracrine signaling and structural components of RGCs: platelet derived growth factor alpha (Pdgfa) and the RGC axon itself. RGC-derived Pdgfa binds to its receptor (Pdgfra) on astrocytes and serves as a mitogen and chemoattractant (Fruttiger et al., 1996; Tao and Zhang, 2016), facilitating migration towards the peripheral retina. While on their migratory route, astrocytes closely associate with RGC axons and are believed to use them as a pre-existing scaffold for motility and spatial patterning. In the retina of mice that lack RGCs (*Atoh7* knockouts) or have RGC axon defects (RGC-specific *Robo1*/*2* mutants), retinal astrocytes display polarization defects, inappropriate migratory patterns and delayed vascular plexus formation (O'Sullivan et al., 2017). Notably, physical RGC-astrocyte interactions are required specifically for directional movement towards the peripheral retina, whereas astrocyte-inner limiting membrane (ILM) interactions are required for any form of astrocyte movement (Gnanaguru et al., 2013; Tao and Zhang, 2016). Thus, RGCs influence the astrocyte lineage – from generation to migration (Fig. 2E,F), and contribute to the development of the retinal vascular landscape.

Following astrocyte migration, endothelial cells are recruited from the optic disc and migrate radially to form a superficial vascular plexus. From this initial plexus, endothelial cells reorient their migration and expand towards the outer retina, forming the deep and intermediate plexus (Fig. 2A) (Sun and Smith, 2018). Beyond RGC-astrocyte interactions, immature retinal neuron activity has also been associated with the development of these vascular plexuses (reviewed by Biswas et al., 2020). Spontaneous activity from retinal neurons, in the form of retinal waves (Huberman et al., 2008), coincides with development of the vascular networks (Weiner et al., 2019). Ablation or chemogenetic inhibition of SACs prevents development of the deep plexus (Weiner et al., 2019). On the other hand, ablating amacrine and horizontal cells severely reduces development of the intermediate plexus (Usui et al., 2015). Although RGCs participate in retinal waves, it has yet to be tested whether their wave-associated activity has a direct effect on vascular development. However, experiments focused on RGC subclasses have hinted towards influence of RGC activity on vascular development.

ipRGCs express melanopsin as early as E15 in the mouse (McNeill et al., 2011), and are one of the earliest established photoreceptors in the neonatal retina (Sekaran et al., 2005; Kirkby and Feller, 2013; Caval-Holme et al., 2019). During vascular development, ipRGCs detect light and suppress angiogenesis, while simultaneously promoting the timely regression of the hyaloid artery (Rao et al., 2013). Visual deprivation (particularly during the fetal period in mice) or deleting melanopsin (*Opn4* knockout mice) leads to persistence of the hyaloid artery and overgrowth of the retinal vascular plexus. Elevated retinal neuron numbers in dark-reared or *Opn4* knockout mice leads to a more hypoxic retina, increased retinal Vegfa production and feedback to the vascular compartment that expresses VEGF receptors (Vegfr2) (Rao et al., 2013; Cristante et al., 2018). In addition, RGCs that express another opsin, *Opn5*, indirectly regulate vascular development (Nguyen et al., 2019). From P3-P8, *Opn5*-RGCs promote light-mediated reuptake of the neurotransmitter dopamine in the neural retina. This activity stabilizes the hyaloid artery by preventing the accumulation of dopamine in the vitreous, where it would act on D2 receptors (Drd2) of endothelial cells to suppress the activity of Vegfr2 (Fig. 2E). Thus, the activity of opsin-expressing RGCs converges on hyaloid artery regression, but acts through distinct mechanisms – one via a Vegfa-Vegfr2 pathway, and the other through a dopamine-D2-Vegfr2 pathway. In support of RGCs as regulators of hyaloid regression, loss of *Atoh7* in mice and humans leads to persistence of the hyaloid artery and failure of the retinal vasculature to effectively develop (Ghiasvand et al., 2011; Edwards et al., 2012; Prasov et al., 2012). Together, the RGC population is a vital signaling and structural hub that establishes the framework for the development of retinal astrocytes and vascular architecture.

### Inter-RGC interactions in the developing visual system

Thus far, we have discussed the various interactions between RGCs and other cell classes in the developing retina. However, RGCs also interact with each other to guide their own development. RGC-to-RGC interactions take place within the retina and at the optic chiasm (where eye-specific axons cross) and appear to be a component of binocular vision development.

**Fig. 3.**
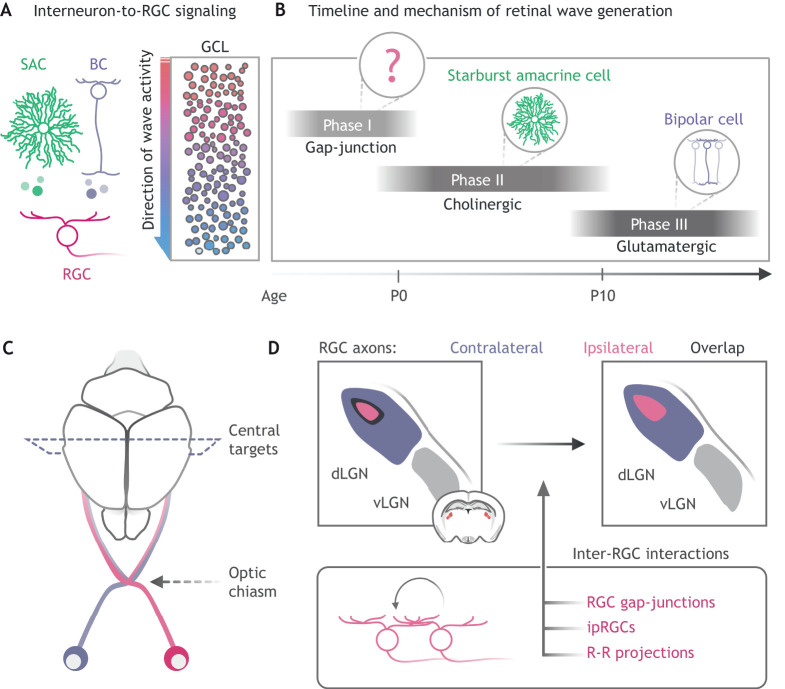
**Inter-RGC interactions shape early retinal activity.** (A) Retinal waves are mediated through release of acetylcholine from starburst amacrine cells (SACs), glutamate via bipolar cells (BC), or through gap-junction coupling between retinal ganglion cells (RGCs). The activity of these cells drives waves of depolarization across the ganglion cell layer (GCL, right) to central targets involved in binocular vision (C,D) and refines synapses between RGCs and their targets. (B) Over developmental time, retinal waves are generated via distinct mechanisms (cell within circle) with partial overlap. Phase I (∼E16-P0), gap-junction-mediated waves; phase II (∼P0-P10), cholinergic waves; and phase III (∼P10-P14), glutamatergic waves. (C) RGCs from each retina extend axons towards the brain and cross at the optic chiasm or continue along the same side (pink versus purple axons). (D) At the visual thalamus (dLGN, dorsal lateral geniculate nucleus; vLGN, ventral lateral geniculate nucleus), retinal waves drive refinement of retinal projections from the eye on the same side (ipsilateral; pink) or opposite side (contralateral; purple). Inter-RGC interactions (bottom) are considered to drive a portion of this refinement process, promoting segregation of projection and thus less overlap (black regions in dorsal LGN). ipRGCs, intrinsically photosensitive RGCs; R-R projections, retino-retinal projections.

During development, spontaneous bursts of synchronized spatiotemporal neuron activity sweep across the retina (termed retinal waves) (Fig. 3A). These correlated bursts of activity are generated through distinct mechanisms over developmental time, and are thought to refine RGC synaptic connections with image-forming brain regions (Fig. 3B; Wong, 1999; Firth et al., 2005; Huberman et al., 2008; Blankenship and Feller, 2010). As a retinal wave sweeps across the ganglion cell layer (GCL), neighboring RGCs fire action potentials followed by a brief refractory period, in which a wave can no longer propagate. This information is received by the visual thalamus and midbrain (McLaughlin et al., 2003; Ackman et al., 2012; Burbridge et al., 2014) and the competition of eye-specific wave activity drives refinement of contralateral and ipsilateral projections (Fig. 3C,D).

RGCs do not passively participate in waves, but rather actively influence eye-specific segregation through diverse mechanisms. The earliest forms of retinal waves (phase I waves) are thought to be propagated by connexin (Cx) gap-junction networks between dendrites of neighboring RGCs (Bansal et al., 2000; Syed et al., 2004) (Fig. 3B). The mechanisms that generate phase I waves are still poorly understood; however, gap-junction propagation plays a role in axon refinement at the dorsal lateral geniculate nucleus (dLGN) of the visual thalamus. Mice that lack gap-junction proteins Cx36 and Cx45 (Cx36/45 double knockout) do not display alterations in phase II or III retinal waves, but have altered inter-wave firing patterns (Blankenship et al., 2011). As a consequence, eye-specific projections in the dLGN are less segregated than wild-type or Cx45 knockout mice. Still, these results warrant further investigation for two reasons. First, Cx36 and Cx45 are both expressed in dLGN neurons, which are recipients of RGC input. Therefore, it is possible that defects observed in the Cx36/45 double knockout arise as a consequence of intrinsic changes at the target rather than within the retina (Belluardo et al., 2000; Maxeiner et al., 2003; Lee et al., 2010; Zlomuzica et al., 2010). Second, amacrine and bipolar cells express Cx36/45 and may influence retinal waves independently of the RGC network (Mills et al., 2001; Han and Massey, 2005; Hansen et al., 2005). Targeted deletion of all detected connexins from RGCs (Cx36, Cx45 and Cx30.2) will provide better insight into the role of inter-RGC gap-junctions in shaping retinal mapping (Völgyi et al., 2009; Müller et al., 2010; Akopian et al., 2014).

During development, ipRGCs also form gap-junction networks with neighboring ipRGCs and conventional RGCs (Kirkby and Feller, 2013; Caval-Holme et al., 2019). Through these intra-retinal gap-junction circuits, ipRGCs increase their own sensitivity to light and simultaneously confer light-sensitivity to conventional RGCs (Caval-Holme et al., 2019). In the absence of phase II retinal waves (β2nAChR knockout and ChAT knockout mice), the retina generates compensatory light-dependent waves that are driven by these ipRGC circuits (Kirkby and Feller, 2013; Stacy et al., 2005). Initially, experiments implicated melanopsin-driven light responses as modifiers of retinal waves and retinal mapping to the dLGN (Renna et al., 2011). More recent analysis, by contrast, suggests that, although ipRGCs participate in phase II waves, their light-dependent activity does not modulate wave properties (Kirkby and Feller, 2013; Chew et al., 2017). M1 ipRGCs, however, appear to be required physically for normal wave properties and maturation of the visual system. Ablation of M1 ipRGCs (*Opn4^DTA^* line) alters properties of phase II retinal waves, leading to failure of eye-specific segregation of retinal-dLGN projections (Chew et al., 2017). As M1 ipRGCs sparsely innervate the dLGN (Hattar et al., 2006; Li and Schmidt, 2018) their interactions likely reflect intra-retinal signaling as opposed to target maturation (ipRGC-to-dLGN axis). Interestingly, M1 ipRGCs project axon collaterals into the inner retina (Joo et al., 2013; Chew et al., 2017) and participate in both dendritic (Tufford et al., 2018) and axon collateral signaling to amacrine cells (Zhang et al., 2008, 2012; Atkinson et al., 2013; Newkirk et al., 2013; Prigge et al., 2016) – thus, these two signaling modalities may explain how ipRGCs shape retinal wave dynamics. Given that the photopigment melanopsin is unnecessary for retinal wave initiation or wave properties, it is still unclear whether activity-dependent or independent mechanisms are employed by M1 ipRGCs to regulate intra-RGC and visual circuit development.

### RGC crosstalk at the optic chiasm shapes binocular vision

Although ipRGCs appear to regulate signaling within the local environment of the retina, some RGCs project their axons to the optic chiasm and into the retina of the other eye (Fig. 3C). These structures, termed retino-retinal or ‘R-R projections’, have been described in amphibians (Bohn and Stelzner, 1981; Humphrey and Beazley, 1985; Tóth and Straznicky, 1989; Tennant et al., 1993), birds (McLoon and Lund, 1982; Thanos, 1999) and mammals (Bunt and Lund, 1981; Braekevelt et al., 1986; Müller and Holländer, 1988; Nadal-Nicolás et al., 2015; Tang et al., 2016). Initially, they were considered non-functional or an experimental artifact, but recent work suggests that R-R projections likely serve a role during development of the visual system. R-R projections have been traced to the GCL of the opposing retina, in which they closely associate with immature SACs (pacemakers of phase II retinal waves) (Fig. 3B; Murcia-Belmonte et al., 2019). These structures likely represent bona fide retinal projections, because the Unc5 axon guidance molecule they express is both necessary and sufficient to generate R-R projections. Although it is difficult to manipulate or record activity of these processes, mathematical modeling suggests that R-R projections may provide inter-retinal synchrony to maximize bilateral eye-specific segregation (Murcia-Belmonte et al., 2019). This is especially true in organisms that have disparate size relationships between the retina and central targets (Murcia-Belmonte et al., 2019). Organisms with similarly sized retina and targets (zebrafish) appear to lack these transient RGC projections and retinal Unc5 expression, primarily relying on molecule-guided retinal mapping and less on retinal waves. Conversely, in mice, *in vivo* recording of retinal waves at the superior colliculus reveals an unexpected level of correlation between waves emerging from both retinae (Ackman et al., 2012), leading authors to speculate that R-R projections functionally synchronize activity in each eye.

R-R projections reflect a small fraction of RGC projections, as the majority direct their axons to central targets. Along their journey from the retina to the brain, axons from both eyes converge at the optic chiasm (Fig. 3C), at which a portion cross over (contralateral or c-RGC) and the remainder maintain their trajectory (ipsilateral or i-RGC). These contra- and ipsilateral-projecting pathways are the basis of binocular vision (Rasband et al., 2003) in vertebrates. The optic chiasm, where RGC axons cross, is a location where axon guidance molecules are interpreted to be repulsive or attractive (reviewed by Erskine and Herrera, 2007; Rasband et al., 2003). This is largely accomplished by interactions between the niche and the intrinsic type of RGC (c-RGC or i-RGC) responding to these cues.

As previously discussed, Shh is expressed by RGCs and acts as a potent mitogen both locally (RPC proliferation) and at the optic disc (astrocyte precursor proliferation). This suggests that it is trafficked down the axon and released at distal locations (Wallace and Raff, 1999; Dakubo et al., 2003). Later in development, Shh is expressed by the earlier-born c-RGC population (Sánchez-Camacho and Bovolenta, 2008), whereas the corresponding Shh receptor Boc is expressed by later-born i-RGCs (Fabre et al., 2010). Given the temporal sequence of birth and axon targeting, it is speculated that c-RGCs influence the targeting of i-RGCs. Consistent with these observations, c-RGCs produce, traffic and deposit Shh in the optic chiasm before the generation of i-RGCs (Peng et al., 2018). As retinal development progresses, i-RGCs project their axon to the chiasm, are repelled by Shh signaling and fail to cross the midline, and thus maintain their ipsilateral trajectories (Peng et al., 2018; Ferent et al., 2019). i-RGC competency to respond to Shh is mediated by expression of the receptor Boc, which is both necessary and sufficient for axon repulsion at the chiasm (Fabre et al., 2010). Taken together, RGCs use multiple mechanisms to shape the development of the binocular visual system – modulation of retinal waves, inter-retinal signaling and regulation of axon guidance allow RGCs to shape communication with central targets before the initiation of vision.

## The roles of RGCs in central target development and behavior

**Fig. 4.**
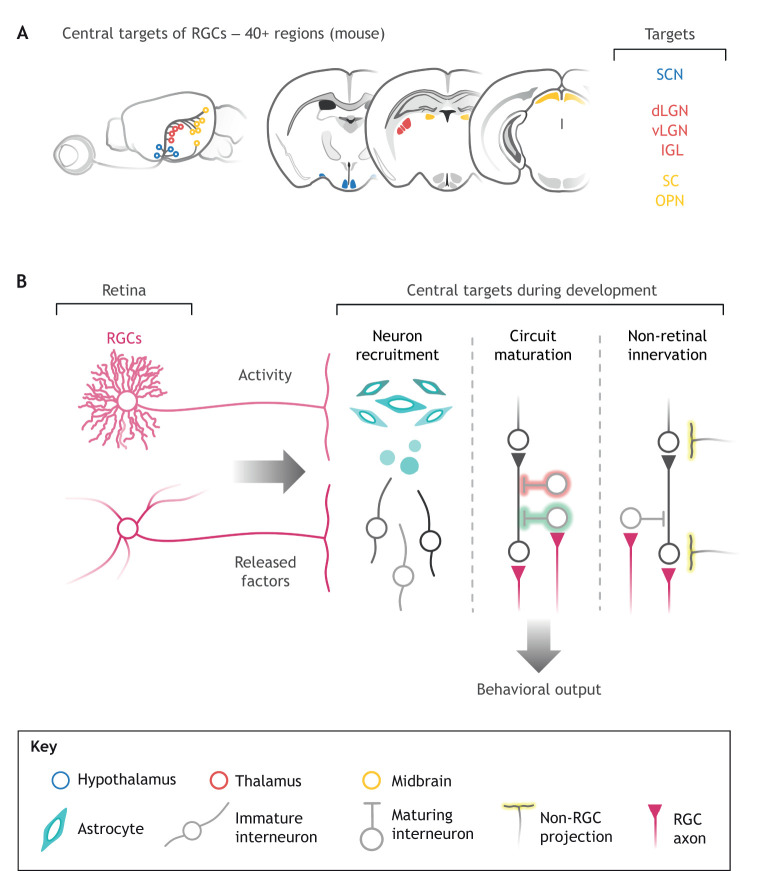
**RGC-regulated central target development.** (A) Projections from the retina terminate in unique central target locations – more than 40 in the mouse brain (Martersteck et al., 2017) – that correspond to regions of the hypothalamus (blue), thalamus (red) and midbrain (yellow). Coronal brain sections highlight major targets of retinal ganglion cells (RGCs) in the hypothalamus (SCN, suprachiasmatic nucleus), thalamus (dLGN, dorsal lateral geniculate nucleus; IGL, intergeniculate leaflet; vLGN, ventral lateral geniculate nucleus) and midbrain (OPN, olivary pretectal nucleus; SC, superior colliculus). (B) RGC projections from the retina influence the recruitment of interneurons via astrocytes, maturation of thalamic circuitry and non-retinal innervation of central targets.

The central portion of the visual system comprises brain regions dedicated to receiving, decoding and dispatching information arriving from the retina. In mice, RGCs project their axons to more than 40 unique regions of the brain (Morin and Studholme, 2014; Martersteck et al., 2017), which we collectively refer to as ‘central targets’. Retinal axons terminate in the hypothalamus, thalamus and midbrain, and thus regulate a variety of behaviors that can be broadly categorized as image forming and non-image forming. In this section, we describe our current understanding of how RGCs shape features of central target cells, influence the innervation of non-retinal inputs and guide the development of behaviors (Fig. 4).

### RGCs in the development of image-forming central targets

As presented in previous sections, RGCs are capable of regulating the development of multiple cell types within a tissue, through diverse mechanisms (activity, secreted factors and structure). This degree of regulation is true for thalamic targets such as the dLGN, in which RGCs synapse onto thalamocortical (TC) relay neurons and inhibitory interneurons. Spontaneous retinal activity, in the form of retinal waves, is largely responsible for the maturation of dLGN circuitry (Failor et al., 2015), and developmental remodeling of TC relay neurons (El-Danaf et al., 2015) and interneurons (Charalambakis et al., 2019). In addition, layer-specific recruitment of dLGN inhibitory interneurons is accomplished by spontaneous activity via RGCs (Golding et al., 2014). RGC axons also provide a physical scaffold and secreted cues to guide development of the dLGN (activity-independent input). Although multiple mechanisms may explain how RGCs recruit interneurons to the appropriate central target, recent transcriptional profiling of the LGN in the absence of retinal input provides some insight into these complex interactions. Within the developing LGN, RGC axons stimulate local astrocytes to produce fibroblast growth factor 15 (Fgf15), which serves as a chemoattractant for newly generated, migrating inhibitory interneurons (Su et al., 2020).

A large portion of input to the visual thalamus consists of axons originating from the cortex (corticothalamic axons), thalamic reticular nucleus and various brainstem nuclei (Guido, 2018). These non-retinal inputs modulate thalamic signaling behavior and are integral to transmission of retinal input to multiple dLGN targets. Given that both retinal and non-retinal axons terminate in the LGN, it is not surprising that both timing and targeting are regulated via axonal interactions. Retinal axons, which arrive at the dLGN early, limit dLGN innervation by corticothalamic axons (Brooks et al., 2013; Seabrook et al., 2013) by inhibiting the degradation of the repulsive chondroitin sulfate proteoglycan (CSPG) aggrecan (Brooks et al., 2013). This axon innervation pathway involves RGC regulation of dLGN interneurons that release aggrecanases (enzymes that break down aggrecan) and serves as a developmental switch to regulate timely innervation of the thalamus. Conversely, projections from the brainstem (ascending cholinergic input) rely on RGCs to promote their timely arrival and axon arborization in the dLGN (Sokhadze et al., 2018). As such, RGCs regulate local events (such as astrocyte signaling and interneuron recruitment) and long-range events (i.e. timely recruitment of corticothalamic and brainstem projections) during thalamic development.

Beyond the visual thalamus, RGCs project axons to other nuclei involved in image forming vision, such as the superior colliculus of the midbrain (SC), as well as nuclei involved in image stabilization, including the medial terminal nucleus (MTN), dorsal terminal nucleus (DTN) and nucleus of the optic tract (NOT). As in the dLGN, complementary ligand-receptor interactions recruit RGC-subclass-specific axons to the MTN and SC (Osterhout et al., 2015; Sun et al., 2015; Ito and Feldheim, 2018). However, whether RGCs influence the development of circuits, non-retinal input or cells within these targets remains to be explored. Given that a single RGC innervates multiple targets (Ellis et al., 2016), and known RGC types that contact the dLGN simultaneously innervate the SC, NOT, DTN and MTN (Kay et al., 2011), it is likely that these central targets all receive similar developmental input from RGCs.

### ipRGCs regulate maturation of the circadian clock and circadian entrainment

The unique ability of ipRGCs to detect photons is matched by their unique projections to central targets. In addition to the visual thalamus, ipRGC axons terminate in multiple nuclei, most notably the suprachiasmatic nuclei (SCN), the intergeniculate leaflet (IGL) and the olivary pretectal nucleus (OPN) (Hattar et al., 2006). These nuclei are collectively termed ‘non-image forming’ because they do not participate in conscious vision; rather, they are involved in behavioral and circadian processes (Fu et al., 2005). As ipRGCs are thought to exclusively express melanopsin, these cells are readily accessed genetically and, therefore, much is known about their developmental influence on non-image forming central targets and their behavioral output.

Throughout the day, rods, cones and melanopsin detect ambient light, which is conveyed via ipRGC axons to the SCN (the circadian pacemaker). Innervation of the SCN via ipRGCs takes place during postnatal life in the mouse (McNeill et al., 2011; Chew et al., 2017), and ipRGCs serve as the primary retinal input to this structure (Hatori et al., 2008; Ecker et al., 2010; Kofuji et al., 2016). This ipRGC-SCN signaling axis allows the organism to synchronize behavior with the light-dark cycle (called photoentrainment). In the absence of an external stimulus or cue, the circadian clock has an endogenous free-running period which is close to – but not exactly – 24 h (Herzog et al., 2017; Hastings et al., 2018). ipRGC activity is necessary for acute entrainment of the circadian clock with the light-dark cycle, but has been shown to also regulate the free-running period of the circadian pacemaker in mice. Removal of the eyes (enucleation) of mice at birth lengthens the circadian period, whereas enucleation into adulthood (P60) has no effect on setting the period, suggesting a crucial window for RGC input to shape SCN output and circadian behavior (Chew et al., 2017). Mice raised in the absence of light or lacking M1 ipRGCs (*Opn4^DTA^* mutants) also have lengthened circadian periods, further implicating light- and activity-dependent ipRGC signaling as a potential mechanism for period setting. Thus, retinal input from ipRGCs is required developmentally, to shape the period length of the clock, and acutely, to synchronize the clock with the light-dark cycle.

M1 ipRGC interactions with the SCN not only set the pace of the circadian clock, but also impact development of non-retinal SCN circuitry. The IGL provides input to the SCN and is involved in nonphotic entrainment of the circadian clock. In the absence of a light-dark cycle, an animal can behaviorally entrain to events that occur at a predictable time across the circadian day, such as time-restricted feeding (Janik and Mrosovsky, 1994; Edelstein and Amir, 1999). ipRGC innervation of the SCN is necessary for the expression of neuropeptide Y (NPY) in IGL neurons projecting to the SCN (Fernandez et al., 2020). Without ipRGC innervation and NPY release at the SCN, the ability to anticipate time-restricted feeding cues is greatly diminished (Fernandez et al., 2020). Together with retinal input-driven thalamic development, these data lead us to reason that RGC input is required to sculpt and shape the intrinsic properties, non-retinal inputs and behavioral output that emerge from central targets in the brain, independently of their adult roles.

## Leveraging transcriptomic and genetic tools to unravel RGC type-specific regulation of visual system development

This Review highlights the developmental roles of RGCs, roles that shape and tune the visual system. As discussed, developmental RGC interactions are diverse and not limited to their adult synaptic partners. As we uncover the diversity of RGCs as a class, a few questions become apparent: what are the contributions of other RGC subclasses and types, if any, to visual system development? Is there a mechanistic bias for certain RGC types within their regulatory roles during development (synaptic, structural or peptide release)?

In the last decade, genetic approaches using Cre recombinase mouse lines that target entire RGC subclasses have provided insight into these questions. This is particularly true for the ipRGC subclass that express *Opn4* and can be accessed through the *Opn4^cre^* mouse line (Ecker et al., 2010). The remarkably diverse influence of ipRGCs on visual system development (Rao et al., 2013; Johnson et al., 2010; Tufford et al., 2018), circadian behavior (Güler et al., 2008; Chew et al., 2017; Fernandez et al., 2020; Hatori et al., 2008), body temperature (Rupp et al., 2019), mood (Fernandez et al., 2018; Huang et al., 2019), learning (Fernandez et al., 2018) and sleep (Rupp et al., 2019) have been assessed using the specificity of *Opn4* expression in combination with Cre-dependent ablation (*Rosa26^iDTR^*) and neuronal silencing alleles (*Rosa26^TeNT^*). It is also clear that, within the ipRGC subclass, distinct RGC types are integral for the development of particular behaviors. These discoveries were made possible through intersectional genetics, with knowledge of RGC type-specific genes (e.g. non SCN-projecting M1 ipRGCs express *Opn4* but not *Brn3b*) and intersectional alleles that allow for recombinase-mediated manipulation of the cell (*Opn4^cre^; Pou4f2^z-DTA^*). It is unlikely that ipRGC types are alone in their roles as developmental regulators. Given that the entire RGC repertoire is established rather early, it is likely that many RGC types and subclasses contribute to development of the retinal and central landscapes.

**Fig. 5.**
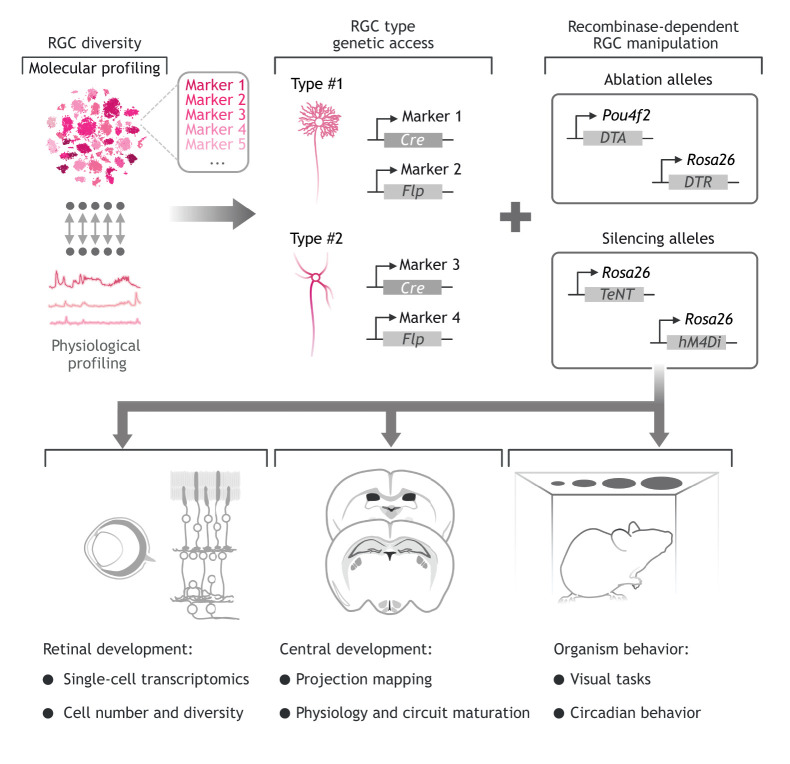
**Deciphering the roles of unique RGC types on the development of the visual system.** To address the fundamental roles of each of the retinal ganglion cell (RGC) types requires a comprehensive understanding of the typology of these cells (RGC diversity). The diversity of RGCs can be defined by their unique transcriptomes (Tran et al., 2019) or electrophysiological responses to visual stimuli (Baden et al., 2016), or a combination of the two. From this, we can define unique combinations of genes that define types of RGCs (e.g. marker 1 and 2 define type #1, and marker 3 and 4 define type #2, etc.) and gain genetic access to these cells by using a combination of recombinase types (e.g. Cre, Flp) driven by subclass-specific genes. Furthermore, genetic access to RGC types would allow us to test whether the cell or its activity is required. Intersectional recombinase-dependent toxin lines (DTA, diphtheria toxin A subunit; DTR, diphtheria toxin receptor) or silencing lines (*hM4Di*, human muscarinic receptor 4 coupled to Gi; TeNT, tetanus toxin) can be applied with RGC type-specific recombinase(s). Finally, as RGCs regulate intra- and extra-retinal development, it will be vital to assess the changes that occur within and outside the retina to elucidate the developmental roles of RGC types.

Recent RGC single-cell RNA-sequencing (scRNA-seq) and physiological profiling studies provide us with a foundation to address RGC type-specific developmental questions (Baden et al., 2016; Rheaume et al., 2018; Tran et al., 2019). Sequencing has revealed candidate marker genes that may exist as recombinase lines (Cre or Flp), which can offer us genetic access to specific RGC types. Although these studies suggest that RGC types can be described by combinations of at least two genes (Tran et al., 2019), dual-recombinase systems are currently implemented to study retinal neuron types in the adult (Jo et al., 2018) and have the potential to be applied to visual system development. Combining recombinase lines (single or dual) with recombinase-dependent ablation/toxin alleles (Mu et al., 2005) or signaling modifying alleles (Huang and Zeng, 2013; Madisen et al., 2015) would allow us to explore the importance of each RGC type and potential activity-dependent or -independent mechanisms on development of the visual system. In addition, temporal control of ablation (*Rosa26^iDTR^*), silencing (*hM4Di*) or activation (*hM3Dq*) can be used to assess when RGC type-specific signaling influences the developmental landscape of the visual system. Considering that the influence of RGCs is not limited to a single cell type within the visual system, it will be important to determine the broader influence of these signaling hubs – particularly through unbiased techniques, such as scRNA-seq, proteomics and behavioral analysis (Fig. 5). Together, scRNA-seq data and genetics may shed light on the emerging developmental roles of RGCs.

## Conclusion

To summarize, we have reviewed the emerging roles of RGCs in the development of the visual system. Along the visual pathway, from retina to targets, we have discussed how RGC activity-dependent and activity-independent inputs regulate the development of diverse cell types within a given niche (neuronal and non-neuronal). Many questions remain to be addressed, such as whether all RGC types are equally poised to regulate development, or which components of vision are a consequence of developmental or adult activity of RGCs. With the derivation of new tools and ‘-omics’ datasets for RGCs, we can begin to determine the RGC-autonomous, non-autonomous and type-specific roles in shaping and establishing the visual system – from molecules and synapses, to circuitry and behavior. Clinically, these forms of integrative multi-system analyses will undoubtedly influence our understanding of how early human RGC loss in conditions such as anophthalmia, primary congenital glaucoma (Wilson and Di Polo, 2012) and *Atoh7* loss-of-function (Prasov et al., 2012; Atac et al., 2019) influence visual system development and behavior beyond conscious vision.
